# Recreational Drug Use among Chinese Men Who Have Sex with Men: A Risky Combination with Unprotected Sex for Acquiring HIV Infection

**DOI:** 10.1155/2014/725361

**Published:** 2014-04-15

**Authors:** Jun-Jie Xu, Han-Zhu Qian, Zhen-Xing Chu, Jing Zhang, Qing-Hai Hu, Yong-Jun Jiang, Wen-Qing Geng, Christiana Meng Zhang, Hong Shang

**Affiliations:** ^1^Key Laboratory of AIDS Immunology of National Health and Family Planning Commission, Department of Laboratory Medicine, The First Affiliated Hospital, China Medical University, Shenyang 110001, China; ^2^Collaborative Innovation Center for Diagnosis and Treatment of Infectious Diseases, Hangzhou, China; ^3^Vanderbilt Institute for Global Health, Vanderbilt University, Nashville, TN, USA; ^4^Division of Epidemiology, Department of Medicine, Vanderbilt University Medical Center, Nashville, TN, USA; ^5^Georgetown University School of Medicine, Washington, DC, USA

## Abstract

*Objective*. To investigate the prevalence of recreational drug use and its relationship with HIV infection among Chinese MSM. *Methods*. A cross-sectional study of 625 MSM was conducted in Shenyang, China. Questionnaires were administered to collect information on recreational drug use and sexual behaviors. Blood specimens were collected to test for HIV and syphilis antibodies. *Results*. Nearly a quarter (23.2%, 145/625) of participants reported ever using recreational drugs, among which alkyl nitrites (poppers) was the most frequently used drug (19.2%), followed by methylmorphine phosphate (5.1%), methamphetamine (4.0%), and ketamine (0.8%). The overall prevalence of HIV and syphilis was 9.6% and 10.4%, respectively. Multivariate logistic analysis showed that recreational drug use was significantly correlated with age ≤25 year (adjusted odds ratio [aOR] = 1.6, 95% CI, 1.1–2.9), single marital status (aOR = 2.1, 95% CI, 1.2–3.6), and seeking male sexual partners mainly through Internet (aOR = 1.8, 95% CI, 1.8–2.8). Recreational drug use was independently associated with an increased risk of HIV infection (aOR = 3.5, 95% CI, 2.0–6.2). *Conclusions*. Our study suggests that recreational drug use is popular among Chinese MSM and is associated with significantly increased HIV infection risk. HIV prevention intervention programs should reduce both drug use and risky sexual behaviors in this population.

## 1. Introduction

Abuse of recreational drugs (also known as club drugs, new-type drugs) [1–3] among men who have sex with men (MSM) has been a worldwide public health concern [1, 2, 4–7]. Compared with traditional illicit drugs (e.g., heroin and marijuana, etc.), recreational drugs are cheaper and easier for distribution in various formats such as liquid, pill, or powder [1, 8]. Recreational drug users can have enhanced feelings of stamina and intoxicating highs and therefore be prone to sexual disinhibition by having more sexual partners or having unprotected anal intercourse (UAI) [5, 9].

With rapid economic development, China and other Asian countries have observed increased popularity of recreational drug use [2, 10–13]. A report published in 2010 showed that 55% of Chinese illicit drug users abused recreational drugs, which exceeded the proportion of abused heroin, a traditionally abused drug in China [3]. Several studies had described the behaviors of using recreational drugs among Chinese MSM. Due to the imbalance of economic development of Chinese cities, the lifetime prevalence of using recreational club drugs varied by city: Chongqing 11.3% [14], Kunming 3.2% [15], Shenyang 1.8% [16], and Heilongjiang 0.3% [17]. A questionnaire survey by mail found that the lifetime average rate of self-reported use of club drugs in six cities was 4.6% [18]. The prevalence seems to be higher among a subgroup of Chinese male sex workers (or money boy). 12% of money boys in Shanghai reported using ecstasy, methamphetamine, or other drugs [19], and 19.9% in Shenzhen used Ketamine, methamphetamine, and so forth [20]. However, few studies have evaluated whether or not recreational drug using behavior was an intrinsic factor impacting the rapid increasing HIV epidemic of Chinese MSM population. It is very necessary to understand the relationship of recreational drug use and HIV infection among Chinese MSM population in order to implement targeted behavioral interventions and active treatment to control their recreational drug using behavior and HIV infection risk [4].

The primary aims of this study are to investigate the prevalence of recreational drug using behaviors, determine factors correlated with recreational drug using behavior, and evaluate the relationship between recreational drug using behavior and HIV infection among MSM in Shenyang, a large inner city in Northeast China.

## 2. Methods and Materials

### 2.1. Study Setting

The study was conducted in Shenyang, a capital city of Liaoning Province in northern China. The HIV epidemic has been rapidly increasing among MSM in Shenyang over the past 5–10 years; for example, the incidence rate was 4.7/100 person-years (PY) in 2007, 6.1/100 PY in 2008, and 10.2/100 PY in 2009 [21, 22].

### 2.2. Participant Enrollment

MSM who were living in Shenyang were recruited to participate in the study in 2011. Snowball sampling methods were used to recruit MSM participants: all MSM were invited through MSM chat rooms by a local gay community-based organization (CBO) called Shenyang Sunny Workgroup to participate in the study. Enrolled participants were asked to refer their friends and sexual partners to participate in the study.

### 2.3. Survey Method and Procedures

The survey was conducted at the Rainbow Harbor voluntary counseling and testing (VCT) Center, which is affiliated with the First Affiliated Hospital of China Medical University. Trained staff at the Rainbow Harbor VCT Center fully explained the study purpose, contents, and procedures at the reception room when MSM came to the clinic. Written informed consent was obtained prior to questionnaire survey. The eligibility criteria were (a) male, self-reporting anal intercourse with male partners in the past 6 months, (b) 18 years or older, and (c) able and willing to provide written informed consent.

### 2.4. Data Collection

Venous blood was drawn by trained nurses after a trained counselor provided pretest counseling. A structured questionnaire interview was administered face-to-face in a private counseling room. The questionnaire included sections on demographics, types, and numbers of sexual partners, condom using conditions with various sexual behaviors in the past three months, alcohol (alcohol users were defined as consuming alcohol at least six times in the past three months), illicit drug use (including recreational drug using behaviors and injection drug using behaviors), and self-perceived HIV infection risk according to each participant's sexual behaviors experience in the past three months (there are four candidate items to choose from: no risk, low risk, medium risk, and high risk).

### 2.5. Laboratory Testing

Sera were tested for HIV and syphilis antibodies. Serum samples were tested for HIV antibody screening using a third-generation enzyme-linked immunosorbent assay (Vironostika HIV-1/2 Microelisa System; BioMérieux, Holland). Samples with positive screening test result were confirmed by HIV-1/2 Western blot assay (HIV Blot 2.2 WB; Genelabs Diagnostics, Singapore). For syphilis testing, serum samples were screened through a rapid regain test (RPR, Diagnosis t; Shanghai Kehua, China). Specimens detected to be RPR positive were confirmed using a Treponema pallidum particle test (TPPA, Serodia, Japan). Participants who were tested positive for both TPPA and RPR were determined to be currently infected with syphilis.

### 2.6. Data Analysis

Data were double entered and checked using Epi-data 3.0 (The Epi Data Association Odense, Denmark, version 3.02) and analyzed using SPSS software (version 17.0; SPSS, Inc., Chicago, IL, USA). Category variables and continuous variables were described by proportions and means, respectively. Logistic regression models were used to assess the factors associated with recreational drug using behavior, such as having multiple (≥3) male sexual partners in the past three months, and HIV infection status. Variables significant at *P* < 0.2 level in the univariate analysis were entered in a multivariate stepwise logistic regression analysis model, and variables significant at *P* < 0.05 level were retained in the final model.

### 2.7. Ethical Considerations

The questionnaire interviews were conducted in private counseling rooms. The study was anonymous, and a unique participant identification number (PID) was used for marking blood specimens and questionnaires. The testing results of HIV and syphilis infections were provided to each participant within one week. Trained staff provided individual posttest counseling. Those with positive HIV testing results were referred to the HIV/AIDS treatment clinic of the study hospital for disease surveillance, care, and support. The study protocol received ethical approval from the Institutional Review Board (IRB) of the First Affiliated Hospital of China Medical University (CMU).

## 3. Results

### 3.1. Characteristics of MSM Using or Not Using Recreational Drugs

A total of 640 MSM were approached for participation in this study. Ten declined and 5 were ineligible due to age <18 years, and the remaining 625 enrolled in the study. Of 625 participants, 145 (23.2%) reported a history of using recreational drugs, in which 120 (19.2%) used poppers (alkyl nitrites or rush), 32 (5.1%) used methylmorphine phosphate (5.1%), 25 (4.0%) used methamphetamine, 5 (0.8%) used Ketamine, 4 (0.6%) used Ecstasy, and 3 (0.5%) used MaGu. Two MSM participants (0.3%) have ever injected drugs. Sixty (9.6%) were HIV positive and 65 (10.4%) were syphilis positive.

Table 1 showed the comparisons of demographic and behavioral characteristics between recreational drug users and nonusers. Compared with nonusers, recreational drug users were more likely to be younger and unmarried, have unprotected anal intercourse (UAI) with regular male sexual partners and multiple (≥3) male sexual partners in the past three months (*P* < 0.05), and seek sexual partners via the Internet. Recreational drug users were less likely to be engaged in UAI with casual male sexual partners in the past 3 months (each *P* < 0.05). There were no statistical differences in current residence, ethnic composition, education level, average monthly income, preferred role in anal sex, commercial sex, and condom use in commercial sex between two groups (*P* > 0.05).

### 3.2. Factors Associated with Use of Recreational Drugs

Table 2 shows the factors associated with use of recreational drugs in univariate and multivariate logistic regression analyses. In univariate analyses, age, marital status, seeking male sex partners via Internet, residence in Liaoning Province, and preferred role in anal sex were significantly associated with use of recreational drug using behavior (*P* < 0.2) and entered to multivariate analysis model. Three factors remained significant in multivariate analysis (*P* < 0.05), and adjusted odds ratio (aOR) and 95% confidence interval (CI) were age ≤25 versus (vs.) >25 years (1.6, 1.1–2.9), single versus married with female or cohabited with males (2.1, 1.2–3.6), and seeking male sex partners via bar/dance halls versus Internet (1.8, 1.8–2.8).

### 3.3. Factors Associated with Having Multiple Male Sexual Partners

A total of 264 (42.2%) participants reported having multiple male sexual partners (≥3) in the past three months.  Table 3 shows the factors correlated with having multiple male sexual partners by univariate and multivariate logistic regression analyses. Educational level, main route of seeking male sexual partners, preferred role in anal intercourse, and ever using recreational drugs were significantly correlated with having multiple male sexual partners (each *P* < 0.2) in univariate analyses. All of these variables were significant in multivariate analysis (*P* < 0.05), namely, educational level of high school and below (versus college or above: 2.0, 1.4–2.9), seeking male sexual partners mainly through Internet (versus mainly through park/public bath: 2.0, 1.1–3.8), predominantly practicing receptive anal intercourse (versus insertive intercourse: 2.1, 1.3–3.4), practicing both insertive and receptive intercourse (versus insertive intercourse: 2.3, 1.5–3.4), and ever using recreational drugs (1.6, 1.1–2.3) (Table 3).

### 3.4. Relationship between Use of Recreational Drugs and HIV Infection

In univariate analysis, the following factors were associated with HIV infection (*P* < 0.05): ever selling sex to male partners, self-perceived high or medium level of HIV risk, no consistent use of condoms with causal male sexual partners, preferring receptive anal intercourse (versus insertive), ever using recreational drugs, and syphilis infection. These variables were included in multivariate analysis. The following variables were retained in the final multivariate logistic regression model as independent predictors for HIV infection: ever selling sex to male sexual partners (3.1, 1.1–9.9), no consistent use of condoms with casual male sexual partners (2.8, 1.4–5.7), ever using recreational drugs (3.5, 2.0–6.2), and syphilis coinfection (3.7, 1.9–7.2) (Table 4).

In univariate analysis, the odds of HIV infection among poppers using MSM were 4.1 times (95% CI, 2.3–7.2, *P* < 0.001) higher than the odds of nonpoppers using MSM participants, and the odds of HIV infection among methamphetamine using MSM participants were 1.8 times (95% CI, 0.6–5.6, *P* = 0.276) higher than the odds of nonmethamphetamine using MSM participants. These factors were not entered for multivariate analysis to avoid the collinearity problems with the recreational drug using behavior variable.

## 4. Discussion

Our study revealed that use of recreational drugs is prevalent among MSM in Shenyang city of China, and poppers and methamphetamine are the most commonly used drugs. Recreational drug use may increase sexual risk behavior and HIV infection [1, 4, 8] and therefore post additional challenge for HIV intervention programs among MSM. The finding also underscores the importance of drug use behavioral surveillance in this population. Public health providers from China's various VCT sites should pay more attention to the collection of recreational drug using behaviors of those clients who seek consultation and HIV testing at VCT sites, whose information may be helpful to conduct targeted behavior intervention to decrease the HIV infection risk of those clients who seek consultation and HIV testing at posttest counseling stage, especially for vulnerable MSM population.

This study expands on the already extensive literature about recreational drug use among MSM in China. The prevalence of drug use among the MSM population in Shenyang, a large northeast city with medium economic level, was higher than in other parts of China [14–17, 23], including male commercial sex workers (money boys) in Shenzhen [23] and Shanghai [19]. The prevalence is similar to that among MSM in Washington D.C. (22.6%) [24] but less than in Sydney (80.3%) [7]. Poppers are the most common drug in this study site, compared to Ketamine in Shenzhen [23], ecstasy in Shanghai [19], and methamphetamine in six other Chinese cities [18]. Among American MSM participants in the Multicenter AIDS Cohort Study, poppers are the most common drug (61%), followed by methamphetamine (23%) and cocaine (14%) [5]. The reasons for the popularity of using popper may include low cost and easy accessibility, as it is widely sold by the Internet retailers in China.

Recreational drug use was associated with younger age, unmarried status, and seeking male sexual partners through the Internet. It was suggested that this subgroup of MSM should be considered a priority targeted group for drug use surveillance and intervention. This study also confirmed that the Internet is the major means of seeking male sexual partners for Chinese MSM [15, 16, 25], and this group is also more likely to use recreational drugs.

Recreational drug using behavior was positively correlated with HIV infection among studied MSM participants, which was in accordance with previous findings among MSM populations in developed countries [5–7, 24, 26]. Recreational drug using MSM had significantly more numbers of multiple male sexual partners in this study, which partly explained their higher HIV infection risk. Previous studies showed that poppers could relax rectal muscles and reduce pain during sex, and therefore use of poppers was associated with increased duration of anal intercourse [1], which can further increase the risk of acquiring HIV. Our study found that poppers using MSM had a 4.1 times greater risk of acquiring HIV than nonusers, which is similar to the finding in the prospective cohort study among American MSM that poppers using MSM had 2.1 times risk of HIV seroconversion [5]. Our study also showed that methamphetamine using MSM had 1.8 times more risk to acquire HIV than nonusers, which was similar to the 2.5 times HIV risk among American MSM [5]. Due to the low prevalence of methamphetamine use, our study did not find a statistically significant relationship between methamphetamine use and HIV infection.

Syphilis can cause genital ulcer diseases (GUDs) and increase HIV infection. Our study showed that syphilis infection was associated with HIV infection, and this finding is consistent with other Chinese studies [15, 16, 21, 27]. It underscores that early testing and timely treatment of syphilis infection should be considered as a measure of HIV prevention.

Our study also confirmed the finding from our previous study that UAI with casual male sexual partners was a risk factor for HIV infection [16]. Although HIV serosorting is considered an effective risk reduction strategy among high risk MSM in the USA [28], this strategy may not be so beneficial for Chinese MSM, because the benefit of this strategy relies on high coverage and frequency of HIV testing [29], while in 2009 only 43.7% of Chinese MSM had ever taken an HIV testing in the past 12 months [30].

Selling sex was independently associated with high HIV risk, and these MSM who sell sex are generally called money boys. Selling sex is often correlated with recreational drug use and a high number of sexual partners [19, 23], both of which may increase HIV risk.

Our study has some strengths. Study participants were recruited and interviewed by trained members of a gay community-based organization that has been our long collaborating partner. We carefully phrased some sensitive questions. For example, instead of asking “whether you once used illicit drugs,” the question is phrased as “whether you once used special substances” to reduce social disability bias.

There are also some limitations. First, the nature of a cross-sectional study prevents concluding any causal relationship between risk factors and HIV infection. Second, the major exposure factor (recreational drug use) was self-reported, the assessment of exposure was weak, and underreporting of the condition was possible in settings where using illegal drugs may be severely punished. Lastly, lifetime recreational drug using behaviors may be different from recreational drug using behaviors immediately prior to having sex with male sexual partners. Research should be continually implemented to further determine the relationships between recreational drug using behaviors, unprotected anal intercourse, and HIV infection status among this vulnerable MSM population.

In summary, recreational drug use is common among Chinese MSM and is associated with having multiple male sexual partners and increased risk of HIV infection. HIV intervention programs targeting MSM should include a component for reducing recreational drug use and its detrimental effects.

## Figures and Tables

**Table 1:** Characteristics of MSM who used or did not use recreational drugs in Shenyang of China.

Characteristics	Drug users (*N* = 145)		Nonusers (*N* = 480)		*χ* ^2^	*P* value
	Number	Proportion (%)	Number	Proportion (%)		
Age, year						
>25	71	49.0	314	65.4	12.741	<0.001
≤25	74	51.0	166	34.6		
Residence in Liaoning Province						
Yes	101	69.7	367	76.5		
No	44	30.3	113	23.5	2.740	0.098
Ethnic						
Han	126	86.9	399	83.1		
Non-Han	19	13.1	81	16.9	1.179	0.278
Educational level						
College and above	59	40.7	176	36.7		
Senior high school and below	86	59.3	304	63.3	0.768	0.381
Marital status						
Married with females/cohabitated with males	19	13.1	136	28.3		
Single	126	86.9	344	71.7	13.850	<0.001
Average monthly income, USD						
≤500	101	69.7	351	73.1		
>500	44	30.3	129	26.9	0.670	0.413
Ever sold sex to male partners						
No	139	95.9	466	97.1		
Yes	6	4.1	14	2.9	0.536	0.464
Main venue to seek male sex partners						
Internet	98	67.6	240	50.0	13.904	0.001
Bar/dance halls	8	5.5	44	9.2		
Park/public bath	39	26.9	196	40.8		
Main sexual role with males						
Receptive anal intercourse	29	20.0	93	19.4		
Receptive/insertive anal intercourse	82	56.6	240	50.0		
Insertive anal intercourse	34	23.4	147	30.6	2.931	0.231
Ever injecting illicit drug						
No	145	100.0	478	99.6		
Yes	0	0.0	2	0.4	0.606	0.436
UAI with regular male partners						
No	103	71.0	381	79.4		
Yes	42	29.0	99	20.6	4.434	0.035
UAI with casual male partners						
No	111	76.6	324	67.5		
Yes	34	23.4	156	32.5	4.312	0.038
UAI with commercial partners in the recent 3 months						
No	137	95.1	454	95.2		
Yes	7	4.9	23	4.8	0.000	0.985
Number of male partners in the past 3 months						
<3	72	49.7	289	60.2		
≥3	73	50.3	191	39.8	5.083	0.024

UAI: unprotected anal intercourse.

**Table 2:** Factors associated with using recreational drugs among MSM in Shenyang of China (*n* = 625).

Factors	*N* (%)	Number of recreational drug use (%)	Univariate analysis		Multivariate analysis	
			OR (95% CI)	*P*	aOR (95% CI)	*P* value
Age, year						
>25	385 (61.6)	71 (18.4)	1.0			
≤25	240 (38.4)	74 (30.8)	2.0 (1.4–2.9)	<0.001	1.6 (1.1–2.9)	0.017
Residence in Liaoning Province						
Yes	157 (25.1)	44 (28.0)	1.0			
No	468 (74.9)	101 (21.6)	0.7 (0.5–1.1)	0.099		
Marital status						
Married with females/cohabitated with males	155 (24.8)	19 (12.3)	1.0			
Single	470 (75.2)	126 (26.8)	2.6 (1.6–4.4)	<0.001	2.1 (1.2–3.6)	0.008
Educational level						
College and above	390 (62.4)	86 (22.1)	1.0			
Senior high school and below	235 (37.6)	59 (25.1)	1.2 (0.8–1.7)	0.381		
Main venue to seek male sex partners						
Park/public bath	235 (37.6)	39 (16.6)	1.0			
Internet	338 (54.1)	98 (29.0)	2.1 (1.4–3.1)	0.001	1.8 (1.2–2.8)	0.005
Bar/dance halls	52 (8.3)	8 (15.4)	0.9 (0.4–2.1)	0.914	1.0 (0.4–2.2)	0.968
Average month income (USD)						
≤500	452 (72.3)	101 (22.3)	1.0			
>500	173 (27.7)	44 (25.4)	1.2 (0.8–1.8)	0.413		
Main sexual role with males						
Receptive anal intercourse	181 (29.0)	34 (18.8)	1.0			
Receptive/insertive anal intercourse	122 (19.5)	29 (23.8)	1.3 (0.8–2.4)	0.295		
Insertive anal intercourse	322 (51.5)	82 (25.5)	1.50 (0.9–2.3)	0.089		

**Table 3:** Factors correlated with having multiple male sexual partners (≥3) among Shenyang MSM by multivariate logistic regression (*n* = 625).

Factors	*N* (%)	Number of having ≥3 male partners (proportion, %)	Univariate analysis		Multivariate analysis	
			OR (95% CI)	*P* value	aOR (95% CI)	*P* value
Age, year						
>25	385 (61.6)	171 (44.4)	1.0			
≤25	240 (38.4)	93 (38.8)	0.8 (0.6–1.1)	0.163		
Residence in Liaoning Province						
Yes	157 (25.1)	62 (39.5)	1.0			
No	468 (74.9)	202 (43.2)	1.2 (0.8–1.7)	0.420		
Marital status						
Married with females/Cohabitated with males	155 (24.8)	65 (41.9)	1.0			
Single	470 (75.2)	199 (42.3)	1.0 (0.7–1.5)	0.929		
Educational level						
College and above	235 (37.6)	76 (32.3)	1.0			
Senior high school and below	390 (62.4)	188 (48.2)	1.9 (1.4–2.7)	<0.001	2.0 (1.4–2.9)	<0.001
Main venues to seek male sex partners						
Internet	338 (54.1)	139 (41.1)	1.0			
Bar/dance halls	52 (8.3)	32 (61.5)	2.3 (1.3–4.2)	0.007	2.0 (1.1–3.8)	0.027
Park/public bath	235 (37.6)	93 (39.6)	0.9 (0.7–1.3)	0.710	0.9 (0.6–1.3)	0.471
Average month income (USD)						
≤500	452 (72.3)	189 (41.8)	1.0			
>500	173 (27.7)	75 (43.4)	1.1 (0.7–1.5)	0.728		
Main sexual role with males						
Insertive anal intercourse	181 (29.0)	53 (29.3)	1.0			
Receptive anal intercourse	122 (19.5)	54 (44.3)	1.9 (1.2–3.1)	0.008	2.1 (1.3–3.4)	0.003
Receptive/insertive anal intercourse	322 (51.5)	157 (48.8)	2.3 (1.6–3.4)	<0.001	2.3 (1.5–3.4)	<0.001
Ever used recreational drugs						
No	480 (76.8)	191 (39.8)	1.0			
Yes	145 (23.2)	73 (50.3)	1.5 (1.1–2.2)	0.025	1.6 (1.1–2.3)	0.027

**Table 4:** Factors associated with HIV infection among MSM in Shenyang of China (*n* = 625).

Factors	*N* (%)	Number of HIV positives (%)	Univariate analysis		Multivariate analysis	
			OR (95% CI)	*P* value	aOR (95% CI)	*P* value
Age, year						
>25	385 (61.6)	30 (7.8)				
≤25	240 (38.4)	30 (12.5)	1.7 (1.0–2.9)	0.054		
Residence in Liaoning Province						
Yes	157 (25.1)	17 (10.8)				
No	468 (74.9)	43 (9.2)	0.8 (0.5–1.5)	0.547		
Ethnics						
Han	100 (16.0)	12 (12.0)				
Non-Han	525 (84.0)	48 (9.1)	1.4 (0.7–2.7)	0.376		
Educational level						
College and above	235 (37.6)	26 (11.1)				
Senior high school and below	390 (62.4)	34 (8.7)	0.8 (0.4–1.3)	0.336		
Marital status						
Married with females/cohabitated with males	155 (24.8)	14 (9.0)				
Single	470 (75.2)	46 (9.8)	1.1 (0.6–2.0)	0.782		
Average month income (USD)						
≤500	452 (72.3)	37 (8.2)				
>500	173 (27.7)	23 (13.3)	1.7 (1.0–3.0)	0.055		
Ever selling sex to male partners						
No	605 (96.8)	55 (9.1)				
Yes	20 (3.2)	5 (25.0)	3.3 (1.2–9.5)	0.025	3.1 (1.0–9.9)	0.05
Main venue for seeking male sex partners						
Internet	235 (37.6)	23 (9.8)				
Bar/dance halls	338 (54.1)	33 (9.8)	1.0 (0.6–1.7)	0.675		
Park/public bath	52 (8.3)	4 (7.7)	0.8 (0.3–2.3)	0.640		
Self-perceived HIV infection risk						
No risk	169 (27.1)	9 (5.3)				
Low risk	264 (42.4)	27 (10.2)	2.0 (0.9–4.4)	0.076		
Medium risk	116 (18.6)	14 (12.1)	2.4 (1.0–5.8)	0.045		
High risk	36 (5.8)	7 (19.4)	4.3 (1.5–12.4)	0.007		
Condom using with casual male partners in the past 3 months						
Consistently used	439 (70.2)	39 (8.9)				
Seldom used	116 (18.6)	7 (6.0)	0.7 (0.3–1.5)	0.325	0.8 (0.3–1.9)	0.601
Never used	70 (11.2)	14 (20.0)	2.6 (1.3–5.0)	0.004	2.8 (1.4–5.7)	<0.001
Preferred role in anal intercourse						
Mainly insertive	181 (29.0)	11 (6.1)				
Mainly receptive	122 (19.5)	16 (13.1)	2.3 (1.0–5.2)	0.039		
Receptive/insertive anal intercourse	322 (51.5)	33 (10.2)	1.8 (0.9–3.6)	0.116		
Number of male sexual partners in the past 3 months						
<3	361 (57.8)	38 (10.5)				
≥3	264 (42.2)	22 (8.3)	0.8 (0.4–1.3)	0.359		
Ever using recreational drugs						
No	480 (76.8)	31 (6.5)				
Yes	145 (23.2)	29 (20.0)	3.6 (2.1–6.3)	<0.001	3.5 (2.0–6.2)	<0.001
Alcohol using behavior						
No	181 (57.3)	37 (20.4)				
Yes	135 (42.7)	20 (14.8)	0.6 (0.3–1.6)	0.649		
Reported male circumcision status						
No	581 (93.0)	54 (9.3)				
Yes	44 (7.0)	6 (13.6)	1.5 (0.6–3.8)	0.349		
Syphilis infection						
No	560 (89.6)	43 (7.7)				
Yes	65 (10.4)	17 (26.2)	4.3 (2.3–8.0)	<0.001	3.7 (1.9–7.2)	<0.001
